# Development and validation of diagnostic KASP markers for MYMIV resistance in urdbean

**DOI:** 10.3389/fgene.2026.1844678

**Published:** 2026-06-15

**Authors:** Debjyoti Sen Gupta, Sachin Kumar, Jitendra Kumar, S. P. Das, Shivam Kumar, A. K. Parihar, Sarbani Banik, J. Souframanien, P. K. Katiyar, G. P. Dixit

**Affiliations:** 1 Division of Crop Improvement, ICAR-Indian Institute of Pulses Research, Kanpur, India; 2 Department of Botany, Chaudhary Charan Singh University, Meerut, India; 3 ICAR Research Complex for NEH Region, Agartala, India; 4 Nuclear Agriculture and Biotechnology Division, Bhabha Atomic Research Centre, Mumbai, India

**Keywords:** blackgram, KASP, molecular marker, MYMIV resistance, SNP, urdbean, *Vigna sp.*

## Abstract

Mungbean Yellow Mosaic India Virus (MYMIV) is a major constraint to productivity in urdbean, necessitating the development of reliable molecular markers for resistance breeding. The present study reports the development of two Kompetitive Allele Specific PCR (KASP) markers for the Tobacco Mosaic Virus (*TMV*) resistance gene and the *F-box/LRR* resistance gene. These markers enable reliable discrimination between resistant and susceptible genotypes in urdbean. The highly MYMIV-resistant genotypes identified in this study possessed resistance alleles for both genes, whereas other resistant genotypes carried the resistance allele for either one of the two genes. This finding broadens the scope of marker-assisted pyramiding of these resistance alleles to enhance the genetic resistance of susceptible or moderately resistant genotypes. Among the urdbean genotypes, most resistant genotypes harboured the resistance allele (GG) for the *TMV* gene. However, only two highly resistant genotypes (IPU11-02 and PU-31) possessed resistance allele for both the *TMV* (GG) and *F-box/LRR* (TT) genes. In mungbean, the susceptible allele (AA) of the F-box/LRR gene was observed in 12 genotypes, while the remaining 12 genotypes exhibited a null allele, indicating the absence of amplification. This allelic distribution, along with the *TMV* gene status, largely explains the comparatively lower degree of genetic resistance to MYMIV in mungbean relative to urdbean. The developed KASP markers were further validated across six additional *Vigna* species. Most genotypes from these *Vigna* species carried the susceptible allele for the *TMV* gene, except PRR 2007-2 of *Vigna umbellata*, which exhibited a high level of resistance to MYMIV under field conditions. For the *F-box/LRR* gene, no alleleic amplification was observed in most genotypes from six *Vigna* species, suggesting the presence of divergent or novel alleles; however, PRR 2007-2 showed amplification with the susceptible allele (AA). In conclusion, the KASP markers developed for the *TMV* and *F-box/LRR* genes provide an efficient and robust tool for marker-assisted introgression of MYMIV resistance alleles. Notably, these markers are effective and applicable in urdbean. These markers can be readily used in breeding programs, including inter-specific crosses involving diverse *Vigna* species, to accelerate the development of MYMIV-resistant cultivars/germplasm.

## 1 Introduction

Urdbean (*Vigna mungo* L. Hepper) and mungbean are among the most important warm-season pulse crops, widely grown in South and Southeast Asia (India, Bangladesh, Pakistan, Nepal, Thailand, and Myanmar) as well as in distant Australia. In India, mungbean production was 2.32 million tonnes (mt) from 3.53 million hectare (mha), while urdbean production was 2.32 mt from 3.53 mha, with average productivities of 598 kg/ha and 656 kg/ha, respectively, during 2023–24 (Pratap, 2025). The productivity of these *Vigna* pulses is adversely affected due to the incidence of multiple biotic and abiotic stresses. Among the biotic stresses, diseases and insect pests causes severe damage, particularly during the rainy season, often resulting in substantial reductions in yield and overall annual production. Mungbean Yellow Mosaic India Virus (MYMIV) is a major impediment in urdbean- and mungbean-growing regions of eastern, central, and northern India (Usharani et al., 2004; Gupta et al., 2022), causing widespread occurrence of yellow mosaic disease in these *Vigna* pulses (Srinivasaraghavan, 2014). Another virus responsible for yellow mosaic disease, Mungbean Yellow Mosaic Virus (MYMV), is more common in the southern and western regions of the country (Karthikeyan et al., 2004). However, studies have demonstrated the occurrence of MYMIV in western and southern peninsular India, suggesting a shift and overlap in the geographoical regions (Reddy et al., 2015; Chowdary et al., 2022). Therefore, considering its wider prevalence and significant impact on pulse production in northern, central, and eastern India, MYMIV was selected as a target pathogen in the present study for improving genetic resistance in *Vigna* pulses.

Our previous study showed that genetic inheritance for MYMIV resistance was due to the presence of oligo-genes in a few urdbean varieties (Sen Gupta et al., 2025; Sen Gupta et al., 2023). The level of MYMIV resistance of the mungbean released varieties in the field is comparatively weaker than that of resistant urdbean varieties. Consequently, mungbean holds further opportunity to improve the degree of genetic resistance in modern varieties by utilizing inter-specific hybridization with resistant urdbean or different other *Vigna* species or wild *Vigna* germplasm. Urdbean and several *Vigna* species (*V. radiata, V. umbellata*, *V. sylvestris*) are infected with MYMIV during crop growth in the absence of genetic resistance. Given that these species are inter-crossable, a reliable molecular marker can be effectively used to identify and select MYMIV-resistant progenies in interspecific hybridization-based breeding programmes.

Previously, MYMIV resistance in urdbean has been reported under the control of single or oligogenes (Gupta et al., 2013; Subramaniyan et al., 2022; Sen Gupta et al., 2023), and a resistance locus was mapped at a distance of 12.8 cM from the closest marker CEDG 180 (Gupta et al., 2013). Later, another marker CEDG116 allowed interval mapping for yellow mosaic resistance (Subramaniyan et al., 2022). Souframanien and Gopalakrishna (2006) developed an ISSR as well as SCAR marker associated with MYMV resistance gene in a set of 53 RILs. On the contrary, monogenic recessive gene regulation and associated SSR markers (CEDG141, CEDG008, CEDG264) were also reported (Sathees et al., 2022). However, the use of these markers could not be adopted routinely in the marker-assisted selection (MAS) for identification of resistant lines in urdbean due to poor association of the markers with MYMV resistance and lack of reproducibility.

Recently, Sen Gupta et al. (2025) reported two resistance genes in urdbean associated with MYMIV, the *Tobacco Mosaic Virus* (*TMV*) resistance gene and the *F-box/LRR* resistance gene, which effectively differentiate between resistant and susceptible genotypes. Through Sanger sequencing, five SNPs were identified in the *F-box/LRR* resistance gene, and a single SNP was detected in the *TMV* resistance gene (Sen Gupta et al., 2025). Based on SNP-445 of the TMV resistance gene, CAPS (Cleaved Amplified Polymorphic Sequence) and TSP (Temperature Switch PCR) markers were developed, and a TSP marker based on SNP-361 was developed for the *F-box/LRR* resistance gene (Sen Gupta et al., 2025). These gene-based functional SNP markers can be effectively utilized for MAS to introgress resistance alleles into elite agronomically superior breeding lines as well as for the genomic selection of resistant haplotypes. However, a more robust, breeder-friendly and high-throughput marker system is required to accurately screen large number of genotypes for MYMIV resistance and susceptibility. In this context, Kompetitive Allele-Specific PCR (KASP) markers represent a powerful molecular tool for the precise detection of SNPs in the genomic DNA. KASP markers are specifically designed to detect DNA polymorphisms between resistant and susceptible genotypes. The underlying principle involves designing allele-specific PCR primers based on known SNP sites, followed by labelling individual alleles with fluorescent dyes such as FAM (Fluorescein) and HEX (Hexachlorofluorescein); FAM is associated with one allele, while HEX is attached to the other. During PCR amplification, the emission of allele-specific fluorescent signals clearly distinguishes the resistant and susceptible genotypes. KASP markers are co-dominant in nature and offer high reproducibility, specificity, high-throughput, and cost-effectiveness. At present, they are the most advanced and efficient molecular marker systems for plant gene mapping, genotyping, and diagnostic applications. Several studies have also demonstrated the successful use of KASP markers in different crop plants including pulses (Slam and Blair, 2018; Mulugeta et al., 2021; Kumar et al., 2024; see Dipta et al., 2024 for review; Kumar et al., 2025).

Inter-specific hybridization is a very commonly used breeding methodology for the development of urdbean and mungbean varieties, especially in India. Genetic control of MYMIV resistance as revealed by the development of KASP markers based on selected SNPs could be used to diagnose resistant and susceptible genotypes in breeding populations, including those emanating from inter-specific crosses involving *V. radiata*, *V. mungo*, *V. sylvestris*, *V. sublobata*, *V. umbellata*, *V. acontifolia,* and *V. glabrescens* besides regular mungbean and urdbean intra-specific crosses. Therefore, the present investigation aimed to develop KASP markers for MYMIV resistance genes applicable to both cultivated varieties and wild *Vigna* germplasm. The primary objective was to develop SNP-based KASP markers capable of distinguishing MYMIV-resistant and susceptible genotypes of urdbean and mungbean based on the sequence variation in the *Tobacco Mosaic Virus* (*TMV*) resistance gene and the *F-box/LRR* resistance gene. Additionally, the developed KASP markers were validated among diverse *Vigna* species, namely, *V. radiata*, *V. mungo*, *V. sylvestris*, *V. sublobata*, *V. umbellata*, *V. acontifolia,* *V. glabrescens* and *V. hainiana*.

## 2 Materials and methods

### 2.1 Gene sequences for KASP marker development

The present experiment was designed to develop molecular markers for the identification of virus-resistant and susceptible urdbean (blackgram) genotypes. From a previously conducted RNA-Seq experiment (PRJNA1174890) (Sen Gupta et al., 2025), two differentially expressed MYMIV resistance genes (*Tobacco Mosaic Virus* resistance gene and *F-box/LRR* gene) were selected for the development of KASP markers, which can distinguish resistant and susceptible genotypes of blackgram (urdbean) and greengram (mungbean). In the previously conducted RNA-seq experiment (Sen Gupta et al., 2025) total clean reads generated from 7.12 to 13.29 GB for the 12 urdbean samples. MYMIV resistant PU-31 and susceptible LBG-17 were grown under infected as well as disease free condition and three biological replications were used for each genotype. Total 563,528 numbers of transcripts were assembled, out of which 553,889 transcripts had coding regions (Sen Gupta et al., 2025). Among these validated transcripts, TRINITY_DN21430_c0_g3_i1 (*Tobacco Mosaic Virus* resistance gene partial sequence) was initially used to design primers which could not be amplified in urdbean DNA. Further, the orthologous gene sequence (XM_014647406.2) of the *Tobacco Mosaic Virus* resistance gene in the *Vigna radiata* (mungbean) genome was used to design primers (Sen Gupta et al., 2025). This primer was used to amplify the DNA of urdbean genotypes, PU-31 and LBG-17. The amplicons were Sanger sequenced, and one SNP (SNP-445)(G to T) was identified (Sen Gupta et al., 2025).

### 2.2 KASP markers validation

#### 2.2.1 Plant materials

A panel of seventy (70) *Vigna* genotypes belonging to eight different species (Table 1) was used to validate the developed KASP markers for two MYMIV resistance genes (*TMV* and *F-box/LRR*). This panel consists of urdbean (*V. mungo*) and mungbean (*V. radiata*) advanced breeding lines and germplasm collections of six other *Vigna* species, including *V. sylvestris*, *V. sublobata*, *V. umbellata*, *V. acontifolia*, *V. glabrescens* and *V. hainiana*. All of these plant materials were raised to seedlings in a greenhouse. After 3 weeks, leaf-tissue samples were collected for DNA extraction using NucleoSpin® Plant II (Takara) Plant DNA extraction kit.

**TABLE 1:** Details of plant materials used in the present study.

Sl.No.	Genotype	*Vigna*_species	Pedigree information
1	PU-19	*V. mungo*	Variety (UPU-1 × UPU-2)
2	PU-31	*V. mungo*	Variety (UPU-97 × DPU88-31)
3	IPU 11–02	*V. mungo*	Variety (DPU88–31 × UC27-2)
4	IPU 10–26	*V. mungo*	Variety (UH85–5 × PDU-103)
5	IPU 13–1	*V. mungo*	Variety (DPU88-31 × UG27-2)
6	KOTA U-4	*V. mungo*	Variety (RBU-38–29 × KUG-489)
7	IC 251383	*V. mungo*	Germplasm collection
8	LBG 752	*V. mungo*	Variety (LBG 402 × LBG 20)
9	LBG 787	*V. mungo*	Variety (LBG 685 × IPU 98–1)
10	LBG 884	*V. mungo*	Variety (LBG 709 × KU 96–3)
11	Uttara (IPU 94–1)	*V. mungo*	Variety (NP 19 × T-9)
12	LBG 904	*V. mungo*	Variety (LBG 645 × TU 94–2)
13	DPU 88–31	*V. mungo*	ABL (PLU-131 × T-9)
14	LBG-685	*V. mungo*	Variety (LBG 402 × NC/CVM)
15	IPU 02–43	*V. mungo*	Variety (DPU88–31 × DUR-1)
16	IPU 18–02	*V. mungo*	Variety (PGRU 95027 × DPU88-31)
17	IPU 21–21	*V. mungo*	ABL^#^ (TV-58 × IPU 02–43)
18	IPU 21–22	*V. mungo*	ABL (yakubpur early × IPU 02–43)
19	IPU 21–23	*V. mungo*	ABL (DPU88–31 × IPU 02–01)
20	IPU 21–24	*V. mungo*	ABL (PU 31 × MASH-114)
21	IPU 21–26	*V. mungo*	ABL (SPS 5 × IPU 02–43)
22	IPU 21–27	*V. mungo*	ABL (DPU88–31 × borda local)
23	IPU 21–29	*V. mungo*	ABL (Shekhar-3 × RUG-216)
24	IPU 21–30	*V. mungo*	ABL (DPU88–31 × IPU 11–06)
25	IPU 21–25	*V. mungo*	ABL (IPU-11–02 × DPU88-31)
26	IPU 21–28	*V. mungo*	ABL (IPU 02–43 × *V. sylvestris*)
27	IPU 21–31	*V. mungo*	ABL (IPU 02–33 × PU-31)
28	IPU 21–32	*V. mungo*	ABL (LBG-623 × MASH-1008)
29	IPU 21–33	*V. mungo*	ABL (IPU 94–01 × *V. sylvestris*)
30	IPU 21–34	*V. mungo*	ABL (IPU02–43 × junagardh local)
31	IPU 21–35	*V. mungo*	ABL (IPU 02–43 × barabanki local)
32	IPU 21–36	*V. mungo*	ABL (IPU 02–43 × STY 2289)
33	IPU 24–1	*V. mungo*	ABL (IPU 02–43 × STY 2289)
34	IPU 24–3	*V. mungo*	ABL (PU-40 × PU-31)
35	IPU 24–13	*V. mungo*	ABL (IPU 94–1 × KU 99–21)
36	IPU 24–15	*V. mungo*	ABL (Shekhar-2 × IPU 11–4)
37	TMB 37	*V. radiata*	Variety (TARM 2 × BM 4)
38	Vasudha (IPM 312–20)	*V. radiata*	Variety (PM3-1 × SPS-5)
39	Varsha (IPM 2 K 14–9)	*V. radiata*	Variety (EC398885 × PDM-139)
40	IPM 02–3	*V. radiata*	Variety (IPM 99–125 × PUSA BOLD -2)
41	Shikha (IPM 410–3)	*V. radiata*	Variety (IPM03–1 × NM-1)
42	Kopergaon	*V. radiata*	Land race
43	Heera (IPM 409–4)	*V. radiata*	Variety (PDM 2881 × IPM 3–1)
44	Kanika (IPM 302–2)	*V. radiata*	Variety (PM 4 × EC 398897)
45	Virat (IPM 205–7)	*V. radiata*	Variety (IPM 02–1 × EC 398889)
46	PUSA 9531	*V. radiata*	Variety (selection from line 9,473)
47	Soorya (IPM 512–1)	*V. radiata*	Variety (IPM99–125 × CO5)
48	IPM 312–4	*V. radiata*	Advanced breeding line
49	IPM 410–9	*V. radiata*	Advanced breeding line
50	IPM 610-2–4	*V. radiata*	ABL (IPM 205–7 × PRR 2008–2)
51	IPM 14–6	*V. radiata*	ABL (PDM 139× ML 729)
52	IPM 701–704	*V. radiata*	ABL (IPM 02–14 × IPU 2–43)
53	IPM 312–394–1	*V. radiata*	ABL (IPM 03–1 × SPS-5)
54	Pant M-5	*V. radiata*	Variety (T44 × UPU-2)
55	IPM 101–102	*V. radiata*	Advanced breeding line
56	IPM 14–49–5	*V. radiata*	Advanced breeding line
57	Meha (IPM 99–125)	*V. radiata*	Variety (PM3 × APM 36)
58	PDM -139	*V. radiata*	Variety (ML 20/19 × ML-5)
59	Kanika (IPM 302–2)	*V. radiata*	Variety (pant M 4 × EC 398897)
60	SML 668	*V. radiata*	Variety (selection from NM 94)
61	PUSA 0672	*V. radiata*	Variety (11/395 × ML 267)
62	LBG-17	*V. mungo*	Variety (netiminmu × chikkuduminu)
63	IC 251427	*V. radiata*	Germplasm line
64	IC 253924	*V. sublobata*	Germplasm line
65	IC 539798	*V. sylvestris*	Germplasm line
66	RBL 1	*V. umbellata*	Variety
67	PRR 2007–2	*V. umbellata*	Germplasm line
68	Wild *Vigna*	*V. aconitifolia*	Germplasm line
69	IC 251381	*V. hainiana*	Germplasm line
70	IC 251372	*V. glabrescens*	Germplasm line

^#^ ABL, advanced breeding line.

### 2.3 Phenotyping for MYMIV disease

All *Vigna* genotypes used in the present study (Table 1) were grown in the research farm of ICAR-IIPR, Kanpur, and their reactions to MYMIV were recorded over the 2 years (2023 and 2024). To ensure uniform disease pressure under natural epiphytotic conditions, the highly susceptible genotype CO-5 was planted after every 10 test genotypes. MYMIV incidence was assessed using a standard disease rating scale presented in Table 2 (Singh et al., 1995). Observations of MYMIV incidence score were recorded at 30–35 days after sowing, when the susceptible check exhibited clear disease symptoms.

**TABLE 2:** Mungbean yellow mosaic india virus (MYMIV) disease rating scale used in the present study.

Score	Disease reaction	Symptoms description
0	Highly resistant	No visible symptoms on leaves (disease-free)
1	Resistant	Very minute yellow specks on leaves
2	Resistant	Small yellow specks covering 0.1%–5% leaf area
3	Moderately resistant	Yellow mottling covering 5.1%–10% leaf area
4	Moderately resistant	Yellow mottling covering 10.1%–15% leaf area
5	Moderately susceptible	Yellow mottling covering 15.1%–30% leaf area
6	Susceptible	Yellow discoloration covering 30.1%–50% leaf area
7	Susceptible	Pronounced yellow mottling; discoloration of seeds/pods; reduced leaf size; stunting (50.1%–75% foliage affected)
8	Highly susceptible	Severe yellow discoloration (75.1%–90% foliage), stunting, reduced pod size
9	Highly susceptible	Severe yellow discoloration (>90% foliage), stunting, no pod formation

### 2.4 SNP genotyping of a selected panel of *Vigna* genotypes

For KASP assays, namely, KASP-TMV and KASP-F-box/LRR, two locus-specific primers and a common reverse primer were synthesized by Europhin, India. Laboratory protocols for the preparation of the KASP assay mix and PCR conditions were used as per the recommendation of LGC Genomics (http://www.lgcgroup.com/). PCR amplification was performed in a 10 µL reaction volume containing 2.0 µL (25 ng/μL) template DNA, 5.0 µL of KASP master mix, 0.14 µL of KASP primer mix, and 2.86 µL ddH_2_O. On each SNP reaction plate, at least one sample was included as a no-template control (NTC). The PCR profile was programmed for an initial denaturation of 94 °C for 15 min, followed by 10 touchdown cycles (94 °C for 20 s, 61 °C–55 °C, decreasing 0.6 °C per cycle for 60 s) and 26 cycles of amplification at 94 °C for 20 s, 55 °C for 1 min, and a final extension of 37 °C for 1 min. Allele discrimination, based on fluorescence data of PCR amplified products, were detected and analyzed using Bio-Rad CFX-96 Real-Time PCR thermal cycler machine.

### 2.5 Data analysis

MYMIV disease incidence scores were recorded for each year (2023 and 2024), and mean values were calculated. Data generated by the Bio-Rad CFX96 system were exported to a spreadsheet, where allele calls were documented. Additionally, SNP discrimination plots were exported as graphical file, depicting the allelic separation of genotypes based on KASP marker genotyping data. The failed samples were excluded during genotype calling and therefore are not visible as a distinct cluster in the plot.

## 3 Results

### 3.1 Development of KASP markers

#### 3.1.1 Disease reaction of the urdbean genotypes used

The urdbean genotype PU-31 is highly resistant to MYMIV disease. As a result of inherent yellow mosaic disease resistance (MYMIV reaction score 0), this variety, after the release in 2005 for Uttarakhand state in India, became one of the mega varieties in urdbean due to its other agronomical, phenological, and other prevalent disease resistance traits, including powdery mildew disease resistance. On the other side, LBG-17 is one of the most popular and powdery mildew-resistant urdbean varieties, which was released in 1985 for the state of Andhra Pradesh in India, and slowly took over vast urdbean areas in peninsular India due to Powdery Mildew (PMW) disease resistance. However, LBG-17 grown under the Kanpur location showed inherent susceptibility to MYMIV disease (MYMIV reaction score 8). Hence, PU-31 and LBG-17 were used as resistance and susceptible sources for KASP marker development, respectively.

#### 3.1.2 Sequencing and marker development

In the present study, partial transcripts of two differentially expressed genes, namely, the *Tobacco Mosaic Virus* (*TMV*) resistance gene and the *F-Box/LRR* genes identified in a previous study of RNA-seq analysis between a MYMIV-resistant urdbean genotype (PU-31) and a highly susceptible genotype (LBG-17) (Sen Gupta et al., 2025), were selected for KASP markers development.

Using this SNP information of *TMV* partial gene sequence, ‘KASP-TMV’ primers were designed. The sequences of the two novel allele-specific forward primers and one common reverse primer are presented in Table 3.

**TABLE 3:** Primer details of KASP-TMV and KASP-F-box/LRR marker.

Marker name	SNP allele	Allele-specific primer 1 (FAM tail)	Allele-specific primer 2 (HEX tail)	Common primer
KASP-TMV	(G/T)	5′GAA​GGT​GAC​CAA​GTT​CAT​GCT​TAT​GAA​CTC​TAA​CAT​GAG​TGG​TGA​G 3′	5′GAA​GGT​CGG​AGT​CAA​CGG​ATTGTT​ATG​AAC​TCT​AAC​ATG​AGT​GGT​GAT 3′	5′CTC​TGC​CAA​TTC​CTT​TGT​CAT​CAT​TTC​AT 3′
KASP-F-box/LRR	(T/A)	5′GAA​GGT​GAC​CAA​GTT​CAT​GCT​GGA​TGA​CCG​TAG​GTA​GAG​TCA​GT 3′	5′GAA​GGT​CGG​AGT​CAA​CGG​ATTGGA​TGA​CCG​TAG​GTA​GAG​TCA​GA 3′	5′CCA​ACA​GAA​CCA​AAG​AAC​AA 3′

Similarly, a primer pair was designed using the partial gene sequence of the *F-box/LRR* gene of urdbean (Sen Gupta et al., 2025). Using this sequence information, ‘KASP-F-box/LRR’ primers were designed. The sequences of the two novel allele-specific forward primers and one common reverse primer are presented in Table 3.

### 3.2 Validation of KASP markers

**FIGURE 1:**
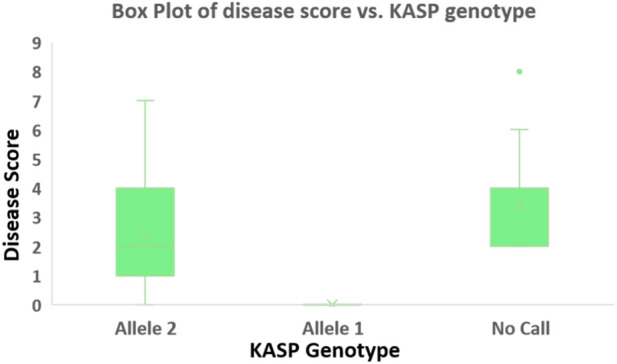
Allelic effect graph or boxplot depicting the disease severity scores on the y-axis grouped by their respective KASP genotypes on the x-axis showing the range of the variability across the tested 70 *Vigna* genotypes.

**FIGURE 2:**
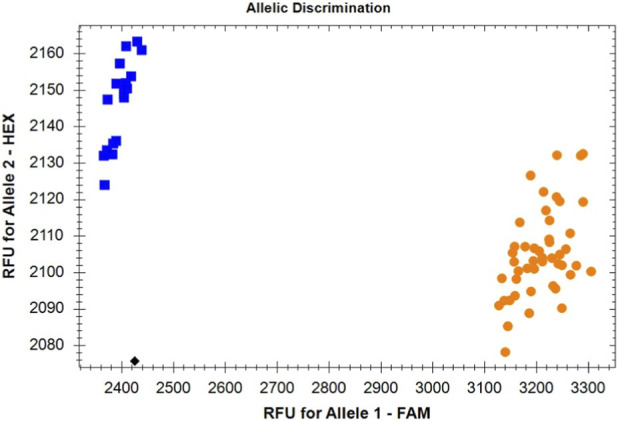
KASP-TMV marker generated orange dots, which represented Haplotype-G (Allele-1) at SNP-445, and blue dots represented homozygous calls for Allele-2 (T), respectively. The Haplotype-G denoted the resistance allele of the *Tobacco Mosaic Virus (TMV)* resistance gene. The fluorescent signal emitted by 47 genotypes (34 urdbeans, 12 mungbeans, and 1 *V. umbellata*) clearly showed them harbouring a resistance allele, G (Table 4; Figure 2). The remaining 18 genotypes (one urdbean, 12 mungbeans, one accession of each of the *V. sublobata, V. sylvestris, V. umbellata, V. acontifolia, V. hainiana*) were homozygous for susceptible T alleles. Black dot at the origin indicates No Template Control (NTC).

**FIGURE 3:**
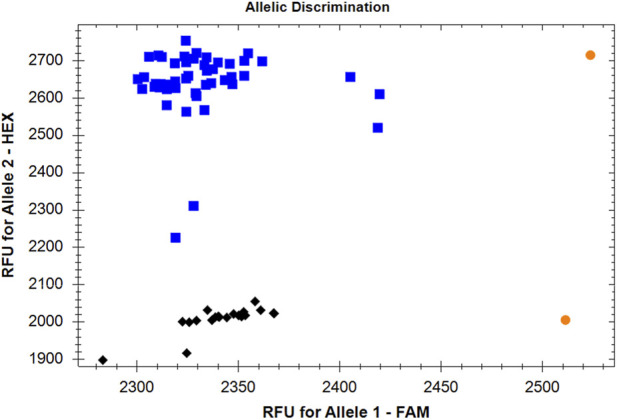
KASP-F-box/LRR marker generated blue dots, which represented Haplotype-A (Allele-2) at SNP-361, and orange represented homozygous calls for Allele-1 (resistance allele T). The Haplotype-A denoted the susceptible allele of the *F-box/LRR* resistance gene. Two genotypes (PU-31 and IPU11-02) had a resistance allele or Allele 1 (T). The fluorescent signal emitted in case of 49 genotypes (34 urdbeans and 14 mungbeans, and PRR 2007-2 (*V. umbellata*)) (Table 4; Figure 3) clearly showed harbouring a susceptible allele, A. The remaining 19 genotypes-*V. radiata* (12), LBG17 (*Vigna mungo*), *V. sublobata* (IC253924), *V. sylvestris* (IC 539798), *V. umbellata* (RBL 1), *V. acontifolia* (Wild *Vigna*), *V. hainiana* (IC 251381), and *V. glabrescens* (IC 251372) had not shown any amplification in KASP analysis.

The validation of developed KASP markers was performed on a panel of 70 genotypes using an RT-qPCR (CFX96 Touch) machine, and the allelic status of these KASP markers with respect to MYMIV resistance genes is presented in Table 4 and Figures 1–3. For the *TMV* gene, the KASP-TMV marker generated orange dots, which represented Haplotype-G (Allele-1) at SNP-445, and blue dots represented homozygous calls for Allele-2 (T), respectively (Table 4; Figure 2). The Haplotype-G denotes the resistance allele of the *Tobacco Mosaic Virus* resistance gene. The fluorescent signal emitted in the case of 47 genotypes (34 urdbeans, 12 mungbeans, and 1 *V. umbellata*) clearly showed them harbouring a resistance allele, G (Table 4; Figure 2). Out of the remaining 23 genotypes eighteen (one urdbean, 12 mungbeans, one accession in each of the other *Vigna* species (*V. sublobata, V. sylvestris, V. umbellata, V. acontifolia, V. hainiana*) were homozygous for susceptible T alleles (Table 4; Figure 2) (Allelic information for five genotypes could not be determined due to failed or ambiguous amplification, and these samples were excluded from clustering in the KASP analysis).

**TABLE 4:** Allelic status of the *Vigna* validation panel with respect to MYMIV resistance genes.

Sl. No.	Name of the genotype used	Name of the *Vigna* species	Allele (*TMV* gene)	Allele (*F-box/LRR* gene)	MYMIV reaction	Disease score
1	PU-31	*V. mungo*	Allele 1	Allele 1	Highly resistant	0
2	LBG-17	*V. mungo*	Allele 2	No call^##^	Highly susceptible	8
3	PU-19	*V. mungo*	Allele 1	Allele 2	Resistant	1
4	IPU 11–02	*V. mungo*	Allele 1	Allele 1	Highly resistant	0
5	IPU 10–26	*V. mungo*	NA^#^	Allele 2	Resistant	2
6	IPU 13–1	*V. mungo*	Allele 1	Allele 2	Resistant	1
7	KOTA U-4	*V. mungo*	Allele 1	Allele 2	Moderately resistant	4
8	IC 251383	*V. mungo*	Allele 1	Allele 2	Resistant	1
9	LBG 752	*V. mungo*	Allele 1	Allele 2	Susceptible	6
10	LBG 787	*V. mungo*	Allele 1	Allele 2	Resistant	2
11	LBG 884	*V. mungo*	Allele 1	Allele 2	Moderately resistant	4
12	IPU 94–1	*V. mungo*	Allele 1	Allele 2	Resistant	1
13	LBG 904	*V. mungo*	Allele 1	Allele 2	Moderately susceptible	5
14	DPU 88–31	*V. mungo*	Allele 1	Allele 2	Highly resistant	0
15	LBG-685	*V. mungo*	Allele 1	Allele 2	Susceptible	7
16	IPU 02–43	*V. mungo*	Allele 1	Allele 2	Resistant	1
17	IPU 18–02	*V. mungo*	Allele 1	Allele 2	Resistant	1
18	IPU 21–21	*V. mungo*	Allele 1	Allele 2	Resistant	1
19	IPU 21–22	*V. mungo*	NA	Allele 2	Resistant	2
20	IPU 21–23	*V. mungo*	Allele 1	Allele 2	Resistant	1
21	IPU 21–24	*V. mungo*	Allele 1	Allele 2	Resistant	1
22	IPU 21–26	*V. mungo*	Allele 1	Allele 2	Resistant	1
23	IPU 21–27	*V. mungo*	Allele 1	Allele 2	Resistant	1
24	IPU 21–29	*V. mungo*	Allele 1	Allele 2	Susceptible	6
25	IPU 21–30	*V. mungo*	Allele 1	Allele 2	Resistant	1
26	IPU 21–25	*V. mungo*	Allele 1	Allele 2	Resistant	1
27	IPU 21–28	*V. mungo*	Allele 1	Allele 2	Resistant	1
28	IPU 21–31	*V. mungo*	Allele 1	Allele 2	Resistant	1
29	IPU 21–32	*V. mungo*	Allele 1	Allele 2	Moderately resistant	4
30	IPU 21–33	*V. mungo*	Allele 1	Allele 2	Resistant	1
31	IPU 21–34	*V. mungo*	Allele 1	Allele 2	Resistant	1
32	IPU 21–35	*V. mungo*	Allele 1	Allele 2	Resistant	2
33	IPU 21–36	*V. mungo*	Allele 1	Allele 2	Resistant	1
34	IPU 24–1	*V. mungo*	Allele 1	Allele 2	Resistant	1
35	IPU 24–3	*V. mungo*	Allele 1	Allele 2	Resistant	1
36	IPU 24–13	*V. mungo*	Allele 1	Allele 2	Resistant	1
37	IPU 24–15	*V. mungo*	Allele 1	Allele 2	Resistant	1
38	TMB 37	*V. radiata*	Allele 1	Allele 2	Moderately resistant	4
39	IPM 312–20	*V. radiata*	Allele 1	Allele 2	Moderately resistant	4
40	IPM 2 K 14–19	*V. radiata*	Allele 1	Allele 2	Moderately resistant	4
41	IPM 02–3	*V. radiata*	Allele 1	Allele 2	Resistant	2
42	IPM 140–3	*V. radiata*	Allele 2	No call	Resistant	2
43	KOPERGAON	*V. radiata*	Allele 1	Allele 2	Moderately susceptible	5
44	IPM 409–4	*V. radiata*	Allele 1	Allele 2	Resistant	2
45	IPM 302–2	*V. radiata*	Allele 1	Allele 2	Moderately resistant	4
46	IPM 205–7	*V. radiata*	Allele 1	Allele 2	Resistant	2
47	PUSA 9531	*V. radiata*	Allele 1	Allele 2	Moderately resistant	4
48	IPM 512–1	*V. radiata*	Allele 1	Allele 2	Moderately resistant	4
49	IPM 312–4	*V. radiata*	Allele 1	Allele 2	Moderately resistant	4
50	IPM 410–9	*V. radiata*	Allele 2	No call	Resistant	2
51	IPM 610-2–4	*V. radiata*	Allele 2	No call	Resistant	2
52	IPM 14–6	*V. radiata*	Allele 2	No call	Resistant	2
53	IPM 701–704	*V. radiata*	Allele 2	No call	Resistant	2
54	IPM 312–394–1	*V. radiata*	NA	Allele 2	Moderately resistant	4
55	PANT M-5	*V. radiata*	NA	Allele 2	Resistant	2
56	IPM 101–102	*V. radiata*	Allele 2	No call	Moderately resistant	4
57	IPM 14–49–5	*V. radiata*	Allele 2	No call	Moderately resistant	4
58	IPM 99–125	*V. radiata*	Allele 2	No call	Moderately resistant	4
59	PDM 139	*V. radiata*	Allele 2	No call	Resistant	2
60	KANIKA	*V. radiata*	Allele 2	No call	Moderately resistant	4
61	SML 668	*V. radiata*	Allele 2	No call	Moderately susceptible	5
62	PUSA 0672	*V. radiata*	Allele 1	Allele 2	Resistant	2
63	​	​	​	​	​	​
64	IC 251427	*V. radiata*	Allele 2	No call	Moderately resistant	4
65	IC 253924	*V. sublobata*	Allele 2	No call	Susceptible	6
66	IC 539798	*V. sylvestris*	Allele 2	No call	Resistant	2
67	RBL 1	*V. umbellata*	Allele 2	No call	Moderately susceptible	5
68	PRR 2007–2	*V. umbellata*	Allele 1	Allele 2	Resistant	0
69	Wild *Vigna*	*V. acontifolia*	Allele 2	No call	Resistant	2
70	IC 251381	*V. hainiana*	Allele 2	No call	Resistant	2
71	IC 251372	*V. glabrescens*	NA	No call	Resistant	2

#NA denotes that this data is not available.

##No call means that there was no amplification of the allele.

For the *F-box/LRR* gene, the KASP-F-box/LRR marker generated blue dots, which represented Haplotype-A (Allele-2) at SNP-361, and orange represented homozygous calls for Allele-1 (resistance allele T) (Figure 3). Two genotypes (PU-31 and IPU11-02) had the resistance allele or Allele 1 (T). The Haplotype-A denotes the susceptible allele of the *F-box/LRR* resistance gene. The fluorescent signal emitted in the case of 49 genotypes [34 urdbeans and 14 mungbeans, and PRR 2007-2 (*V. umbellata*)] (Table 4; Figure 3) clearly showed harbouring a susceptible allele, A. The remaining 19 genotypes, *V. radiata* (12), LBG17 (*Vigna mungo*), *V. sublobata* (IC253924), *V. sylvestris* (IC 539798), *V. umbellata* (RBL 1), *V. acontifolia* (Wild *Vigna*), *V. hainiana* (IC 251381), and *V. glabrescens* (IC 251372), had not shown any amplification in KASP analysis. (Table 4; Figure 3).

### 3.3 MYMIV disease reaction and allelic status

Susceptible reactions was significantly higher than resistant reactions in the tested panel of 70 *Vigna* genotypes (p < 0.001) as reflected by their respective disease severity scores. There was one urdbean genotype (LBG-17) which exhibited a highly susceptible disease reaction (Score 8). There were three moderately resistant urdbean genotypes, Kota Urd −4, IPU21-32, and LBG-884 (Score 4). LBG-904 exhibited a moderately susceptible disease reaction (Score 5). There were three urdbean genotypes which were susceptible to MYMIV infection, LBG-752, LBG-685, and IPU21-29 (Score 6 or 7). Out of the 37 urdbean genotypes, 28 exhibited resistant reaction. Among these 28 resistant genotypes, three genotypes, i.e., PU-31, IPU11-02 and DPU88-31 were highly resistant (Score 0) and the remaining 25 urdbean genotypes were resistant, IPU21-25, IPU21-22 and IPU10-26 (Score 2), IC 251383, PU-19, IPU94-1, IPU02-43, IPU18-02, IPU21-21, IPU21-22, IPU21-23, IPU21-24, IPU21-26, IPU21-27, IPU21-30, IPU21-25, IPU21-28, IPU21-31, IPU21-33, IPU21-34, IPU21-36, IPU24-1, IPU24-3, IPU24-13, IPU24-15 and IPU13-1 (Score 1). Most of the tested resistant genotypes (34 out of 37) of urdbean in the present investigation harbored the resistance allele (G) for the *TMV* gene, and among them, only two genotypes (PU-31 and IPU11-02) also carried the resistance allele (T) for the *F-box/LRR* gene besides the resistance allele of the *TMV* gene (Table 4).

Among the 26 tested mungbean genotypes, 11 genotypes showed a resistant reaction (Score 2)- IPM 02-3, IPM 140-3, IPM 409-4, IPM 205-7, IPM 410-9, IPM 610-2-4, IPM 14-6, IPM 701-704, Pant M 5, PDM 139, and Pusa 0672. Besides this, 13 mungbean genotypes were moderately resistant: TMB 37, IPM 312-20, IPM 2K 14-19, IPM 302-2, Pusa 9531, IPM 512-1, IPM 312-4, IPM 312-394-1, IPM 101-102, IPM 14-49-5, IPM 99-125, Kanika, and IC 251427. Two genotypes, Kopergaon and SML 668, exhibited a moderately susceptible reaction to MYMIV infection. In these genotypes, 12 genotypes had the resistance allele (G) and another 12 genotypes had the susceptible allele (T) for the *TMV* gene. While for the remaining 2 genotypes data could not be generated. However, the susceptible allele (A) of the *F-box/LRR* gene was only amplified in 14 genotypes, while the remaining 12 mungbean genotypes showed no call of either allele of the *F-box/LRR* gene (Table 4).

Among the other *Vigna* species, *V. glabrescens* (IC 251372), a mothbean genotype (Wild *Vigna)* (*V. acontifolia*), and the IC 251381 genotype of *V. hainiana* showed a resistant reaction (Score 2). Among the 2 *V. umbellata* genotypes tested here, one was resistant, and the other was susceptible. PRR 2007–2 was highly resistant (Score 0), and RBL 1 was moderately susceptible (Score 5). One *V. sylvestris* was found to be resistant (Score 2), and 1 *V. sublobata* (IC 253924) showed a susceptible reaction (Score 6). In these genotypes, only a genotype (PRR 2007–2) of *V. umbellata* carried the resistant allele (G) of the *TMV* gene, while the remaining genotypes of other species (Table 4) had either the susceptible allele (T) or no amplification of this gene. However, only a genotype (PRR 2007–2) of *V. umbellata* carried a susceptible allele of the *F-box/LRR* gene, while other genotypes had no call for the *F-box/LRR* gene (Table 4).

## 4 Discussion

Mungbean yellow mosaic India virus (MYMIV) poses a severe threat to mungbean and urdbean cultivation, leading to substantial yield losses across tropical and subtropical regions (Karthikeyan et al., 2014; Rathi, 2002). In the present study, we validated the KASP markers developed for *TMV* and *F-box/LRR* genes for MYMIV-resistant genotypes. Our results showed that most of the tested resistant urdbean genotypes (34/36) carrying the resistance allele (G) for the *TMV* gene have a disease reaction score of 0–7 and while two resistant genotypes (PU-31 and IPU11-02) carrying the resistance allele (T) of *F-box/LRR* gene and resistant allele (G) of *TMV* gene have disease reaction score of ‘0’. These results indicate that this gene is enhancing the MYMIV resistance in urdbean. However, one urdbean genotype DPU 88–31 had a disease reaction score of ‘0’ and carried only the resistant allele (G) of the *TMV* gene and susceptible allele (A) of the *F-box/LRR* gene, indicating that some other genes are also present in this genotype, which are responsible for enhancing the MYMIV resistance in urdbean. Thus, our results are in contrast with earlier studies that single or oligogenes control MYMIV resistance in the urdbean (Sen Gupta et al., 2023; Subramaniyan et al., 2022; Sathees et al., 2022; Gupta et al., 2013).

In the present investigation, several mungbean genotypes having resistance, moderately resistant, and susceptible disease reaction carried the resistance allele (G) of *TMV* genes and either the susceptible allele or no amplification of the *F-box/LRR* gene, indicating no role of the *F-box/LRR* gene in contributing resistance to MYMIV and the presence of other genes controlling MYMIV resistance in mungbean. An earlier report also identified a major QTL on chromosome 4, designated as *qMYMV4-1*, that harboured several possible candidate genes for controlling MYMV resistance (Mathivathana et al., 2019).

It was also seen that in the case of many genotypes from different *Vigna* species including *V. sublobata, V. sylvestris, V. acontifolia, V. glabrescens* and *V. hainiana* there was no amplification of the *F-box/LRR* gene. One (PRR 2007–2) of the genotypes of *V. umbellata* carries a susceptible allele (T) of the *F-box/LRR* gene along with a resistance allele (G) of the *TMV* gene, under field conditions exhibiting a highly resistant disease reaction. Another moderately susceptible genotype of *V. umbellata* (RBL-1) harbored a susceptible allele (T) of the *TMV* gene and no amplification of the *F-box/LRR* gene. However, RBL-1 of *V. umbellata* was reported as a resistant source for MYMIV resistance in an earlier study and identified a large-effect QTL conferring MYMIV resistance introgressed from RBL-1 of *V. umbellata* in the urdbean variety Mash114 (Dhaliwal et al., 2022). Thus, our study, in fact, showed that *V. umbellata* could be a potential source of MYMIV resistance in addition to its agronomic traits and abiotic stress tolerance attributes; however, caution should be practiced to select MYMIV-resistant genotypes for trait transfer through inter-specific hybridization. It is evident from the present investigation that there are potential sources of genetic resistance for MYMIV disease, and gene pyramiding is possible even using marker-assisted selection when disease pressure is not high in the greenhouse or field facilities due to climatic or agronomic conditions.

Inter-specific hybridization is very common in urdbean and mungbean breeding programmes (Bhardwaj et al., 2022). In a recent study, about 1163 interspecific crosses among urdbean and wild *Vigna* species were attempted (Umdale et al., 2020). The maximum crossability was recorded between urdbean and its wild progenitor, *V. silvestris*. In addition urdbean was successfully crossed with *V. radiata, V. hainiana, V. sublobata, and V. stipulacea* (Umdale et al., 2020). Hence, identification of MYMIV-resistant lines in the interspecific populations has become possible through the use of the newly developed KASP markers. This may lead to the broadening of the genetic base without loosening inherent MYMIV resistance in a variety.

The recent next-generation sequencing of most pulse crops generated an enormous quantity of genomic resources, including the availability of molecular markers like SNPs, SSR, and INDELs, which are usually used for fine mapping of disease resistance genes (Dhole et al., 2024). Using such marker-assisted selection in Chickpea, new varieties like Pusa Chickpea-10216 and Super Annigiri-1 were developed (Dhole et al., 2024). In urdbean and mungbean, such advancements are now possible for MYMIV disease resistance introgression using marker-assisted selection.

In the present study, PU-31, IPU11-02, and DPU88-31 were highly resistant, and they can be routinely used in urdbean and mungbean breeding programmes as donors for both genes, *TMV* and *F-box/LRR* resistance genes. It is imperative to note that the MYMIV disease resistant urdbean varieties developed by ICAR-Indian Institute of Pulses Research, Kanpur, and released for cultivation during the past years are very high and have been identified as highly resistant with a score of ‘0’ (IPU11-02), resistant with a Score of ‘2’ (IPU10-26), and with a Score of ‘1’ (IPU94-1, IPU02-43, and IPU13-1). This is one of the reasons, besides agronomic preferences and high-yield, that these urdbean varieties are very popular among the urdbean farmers, as three highly MYMIV resistant varieties IPU11-02, IPU13-1, and IPU10-26 have a share 25.11% in the total breeder seed indent of urdbean in the country (Annual Report on Mungbean et al., 2024).

In conclusion, these markers can reliably identify resistant and susceptible urdbean varieties/genotypes. In the case of highly MYMIV-resistant genotypes, resistance alleles for both genes were found, and in the rest of the cases, either of the resistance alleles was present, which further broadens the scope of marker-assisted pyramiding of these resistance alleles in susceptible or moderately resistant genotypes to elevate their genetic resistance level. These two sets of KASP markers will be helpful in marker-assisted introgression of MYMIV resistance alleles in future urdbean breeding programmes.

## Data Availability

The original contributions presented in the study are publicly available. Raw reads of the RNA-Seq experiment (Sen Gupta et al. 2025) which was the data source of the current study can be found in NCBI (BioProject Id: PRJNA1174890); partial gene sequences of putative TMV and F-box-LRR genes can be retrieved from Sen Gupta et al. 2025. Further inquiries can be directed to the corresponding authors.
